# Impact of a computerized system for evidence-based diabetes care on completeness of records: a before–after study

**DOI:** 10.1186/1472-6947-12-63

**Published:** 2012-07-07

**Authors:** Pavel S Roshanov, Hertzel C Gerstein, Dereck L Hunt, Rolf J Sebaldt, R Brian Haynes

**Affiliations:** 1 Schulich School of Medicine and Dentistry, The University of Western Ontario, 1151 Richmond Street, London, ON, Canada; 2 Department of Medicine, McMaster University, 1280 Main Street West, Hamilton, ON, Canada; 3 Hamilton Health Sciences, 1200 Main Street West, Hamilton, ON, Canada; 4 Health Information Research Unit, Department of Clinical Epidemiology and Biostatistics, McMaster University, 125 Communications Research Laboratory, 1280 Main Street West, Hamilton, ON, L8S4K1, Canada

## Abstract

**Background:**

Physicians practicing in ambulatory care are adopting electronic health record (EHR) systems. Governments promote this adoption with financial incentives, some hinged on improvements in care. These systems can improve care but most demonstrations of successful systems come from a few highly computerized academic environments. Those findings may not be generalizable to typical ambulatory settings, where evidence of success is largely anecdotal, with little or no use of rigorous methods. The purpose of our pilot study was to evaluate the impact of a diabetes specific chronic disease management system (CDMS) on recording of information pertinent to guideline-concordant diabetes care and to plan for larger, more conclusive studies.

**Methods:**

Using a before–after study design we analyzed the medical record of approximately 10 patients from each of 3 diabetes specialists (total = 31) who were seen both before and after the implementation of a CDMS. We used a checklist of key clinical data to compare the completeness of information recorded in the CDMS record to both the clinical note sent to the primary care physician based on that same encounter and the clinical note sent to the primary care physician based on the visit that occurred prior to the implementation of the CDMS, accounting for provider effects with Generalized Estimating Equations.

**Results:**

The CDMS record outperformed by a substantial margin dictated notes created for the same encounter. Only 10.1% (95% CI, 7.7% to 12.3%) of the clinically important data were missing from the CDMS chart compared to 25.8% (95% CI, 20.5% to 31.1%) from the clinical note prepared at the time (*p* < 0.001) and 26.3% (95% CI, 19.5% to 33.0%) from the clinical note prepared before the CDMS was implemented (*p* < 0.001). There was no significant difference between dictated notes created for the CDMS-assisted encounter and those created for usual care encounters (absolute mean difference, 0.8%; 95% CI, −8.5% to 6.8%).

**Conclusions:**

The CDMS chart captured information important for the management of diabetes more often than dictated notes created with or without its use but we were unable to detect a difference in completeness between notes dictated in CDMS-associated and usual-care encounters. Our sample of patients and providers was small, and completeness of records may not reflect quality of care.

## Background

Successful diabetes care requires the active participation and informed self-management of engaged, educated, and motivated patients. The healthcare team, in turn, requires timely access at the point of care to comprehensive patient information, including but not limited to weight, blood pressure, current medications, glycosylated haemoglobin (HbA1c), serum creatinine, LDL cholesterol, and urine albumin to creatinine ratio. Easy access to this information helps clinicians and patients to tailor and optimize care. Collection of standardized and well-defined data on each patient also allows for provider-level and clinic-level summary data for quality improvement.

An electronic, diabetes-specific, chronic disease management system (CDMS) that collects limited data during the clinical encounter and presents these data during future clinical encounters may help meet the needs of diabetic patients and their healthcare team.

In a series of 6 systematic reviews covering 166 randomized controlled trials (RCTs), we recently synthesized the evidence of effectiveness of computerized clinical decision support systems for primary prevention [1], diagnostic test ordering [2], acute care [3], drug prescribing and management [4], therapeutic drug monitoring and dosing [5], and management of chronic diseases [6]. Several systems for management of diabetes have been tested in RCTs and a small majority improve practitioner performance, with less success for enhancing patient outcomes [7-25].

However, creating effective clinical information systems remains difficult. Because most systems are developed and tested in a few highly computerized environments with a long history of informatics excellence, the findings may not be generalizable to the more technologically naïve settings where most people receive care. Recent attention on health information technology as a means for better healthcare has made it increasingly important to design and test systems in a variety of settings.

### The P-PROMPT CDMS in the Diabetes Care and Research Centre

With the help of diabetes specialists, Fig.P Software Inc. tailored its web-based chronic disease management system, the P-PROMPT CDMS, specifically for diabetes care and deployed it in the Hamilton Health Sciences Diabetes Care and Research Centre (DCRC), a hospital-based, ambulatory care subspecialty clinic. The system’s record structure is based on clinical practice in the DCRC and on the Canadian Diabetes Association practice guidelines for 2008, updated by us to 2010 to reflect more recent evidence. In a typical patient visit to the DCRC, physicians interview and examine patients in a room with an internet-connected computer. They always dictate a note summarizing the patient visit. Some practitioners tend to dictate their summary immediately after the patient leaves, while others defer this until the end of the day. Once transcribed, this dictated note is stored in the patient’s paper records and communicated to their family doctor. Prior to the DCRC’s recent adoption of the CDMS, this note, and any laboratory test results attached to it, comprised the longitudinal record of each patient.

**Figure 1:**
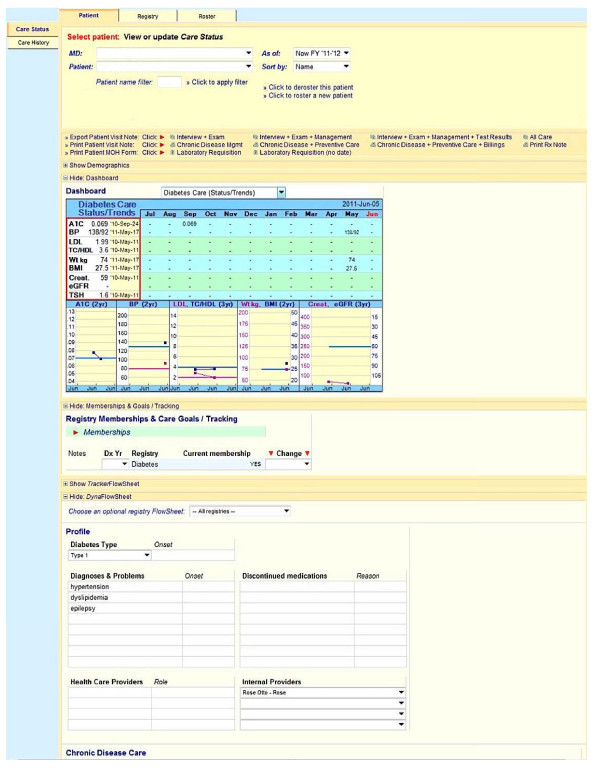
CDMS dashboard.

**Figure 2:**
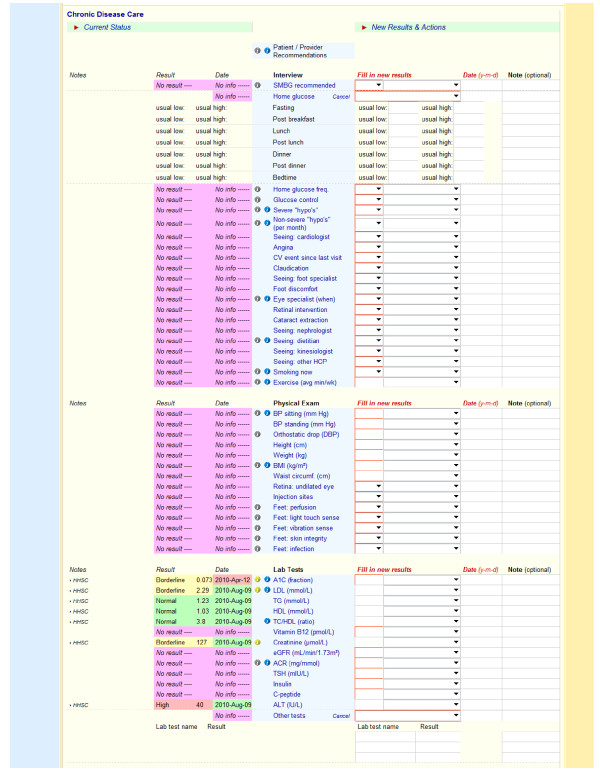
CDMS diabetes checklist.

**Figure 3:**
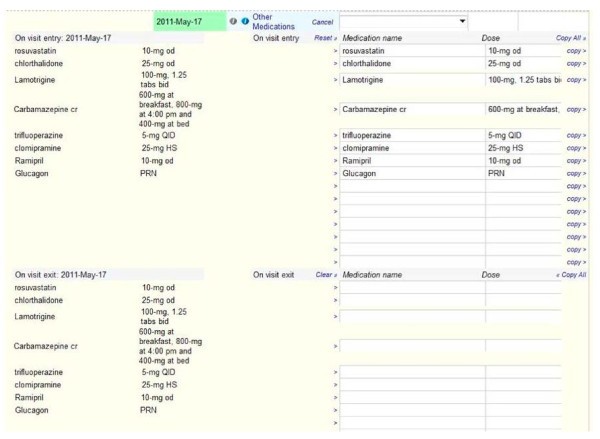
CDMS medications.

Figures 1, 2, 3 show screenshots of the CDMS interface. During or after a patient visit, physicians enter directly into the CDMS information important for the management of diabetes, including each patient’s demographic information, existing diagnoses, disease registry membership, and complete medication profile that includes current and past medications, along with reasons for discontinuation. The system is linked to the hospital’s electronic laboratory records and summarizes the results of laboratory tests in a *dashboard* that displays longitudinal trends graphically; results from tests performed outside of the hospital must be entered manually. Finally, the CDMS can provide point-of-care, guideline-based, patient-specific decision support for practitioners and tailored self-management support messages intended for patients.

Before carrying out a large study to test the impact of the CDMS on diabetes care in the DCRC, we conducted a small pilot to help with sample size calculations. For this study we asked, “Does the CDMS impact the recording of clinical information pertinent to evidence-based diabetes care?”

## Methods

**Figure 4:**
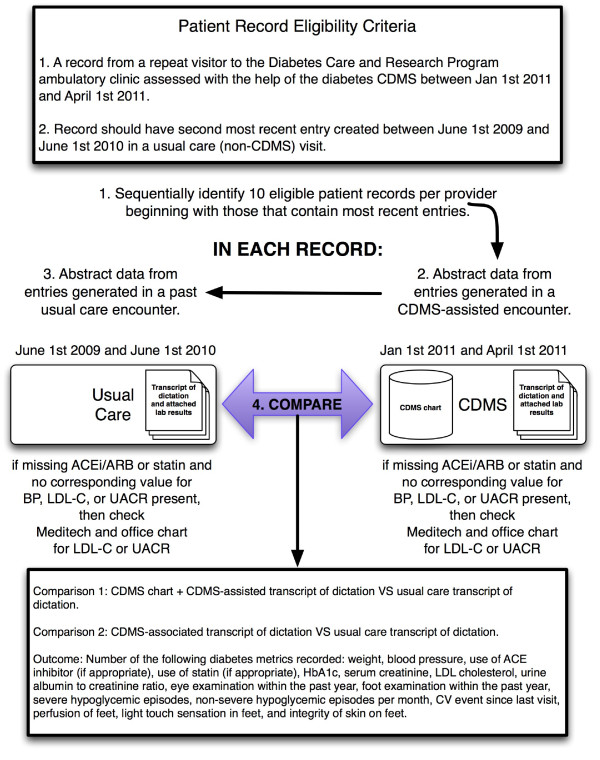
**Study design.** CDMS, Chronic disease management system; BP, Blood pressure; ACEi, Angiotensin Converting Enzyme inhibitor; ARB, Angiotensin II Receptor Blocker; HbA1C, Glycosylated haemoglobin; Cr, Creatinine; LDL, Low density lipoprotein cholesterol; UACR, Urinary albumin to creatinine ratio; CV, Cardiovascular.

Figure 4 summarizes the study design. This was a retrospective chart review of recent patient record entries (electronic charts and transcripts of dictated notes with any attached laboratory reports) generated as a result of CDMS-associated visits and previous transcripts of dictated notes (and attached laboratory reports) created without the help of the CDMS for the same patients. The Hamilton Health Sciences Research Ethics Board provided ethics approval for this study.

### Eligibility of patient records

Patient records were eligible for review for patients who had their first complete CDMS-associated assessment at the clinic between January 1st 2011 and April 1st 2011 and previously without the help of the CDMS between June 1st 2009 and June 1st 2010, with no intervening DCRC physician visit (ignoring visits made to other health professionals).

### Primary outcome

16 items of particular interest included the patient’s weight; blood pressure; use of statins when appropriate; use of Angiotensin Converting Enzyme (ACE) inhibitors or Angiotensin II Receptor Blockers (ARBs) if appropriate; HbA1c; serum creatinine; LDL cholesterol; urine albumin to creatinine ratio (UACR); eye and foot examinations within the past year; any occurrence of severe hypoglycaemic episodes; number of non-severe hypoglycaemic episodes per month; cardiovascular events since the previous visit; and perfusion, light touch sensation, and skin integrity of the feet.

We counted the number of items in each record and calculated a *missing fraction* using Formula 1

(1) $$Missing\;Fraction=\left(\frac{16-\#present}{16}\right)\times 100$$

For example, a record with 12 present items would have a missing fraction of 25%. We found the mean missing fraction by first finding the missing fraction for each patient’s records and calculating their mean. We considered items to be *present* if clearly stated in the record or not relevant for a given patient. We used values for blood pressure and UACR to judge whether a patient’s use of an ACE inhibitor or ARB should have been noted in that patient’s record, as the relevance of these medications depends on those values. ACE inhibitors or ARBs are appropriate if the UACR is greater than 2.0, or if the patient is known to be hypertensive or blood pressure measured in clinic is above 130/80 mmHg. Statin medications are appropriate if LDL cholesterol levels exceed 2.0 mmol/L, if a diabetic patient is male and over 45 years of age or female and over 50 years of age, or if the patient has ever experienced a stroke, myocardial infarction, or other major vascular event.

Our outcome of interest was the mean absolute difference in missing fraction between pairs of record types (see Analysis). Note that the CDMS does not directly assist dictation. *CDMS-associated dictation* refers to dictated notes that correspond to patient visits in which the CDMS was used.

### Measurement protocol

From a larger group of 7 physicians in the DCRC, we selected for participation in this pilot study 3 attending physicians with a special interest in medical informatics. First, each clinician screened his patients’ records sequentially for inclusion according to the eligibility criteria, beginning with the record updated most recently and moving to records updated progressively earlier until 10 eligible patient records were identified. Second, documentation pertaining to 2 separate clinical encounters was assessed within each record: the documentation generated during the recent CDMS-associated encounter and that which was generated during the next most recent encounter that did not include the CDMS, for a total of 61 encounters documented in 31 patient records.^a^

Records were assessed in two phases: 1) review of all dictated notes and 2) review of electronic charts from the CDMS (depending on whether the CDMS was used or not) created during the corresponding patient encounter. We ordered these review phases to minimize any impact of the CDMS chart assessment on assessment of the dictated note.

### Analysis

We estimated the mean differences and standard deviations between patient records for the proportion of missing items. We assessed 3 different record types for each patient (transcript of dictation from visit in which the CDMS was used [CDMS-associated transcript], transcript of dictation from usual care [non-CDMS] visit, and CDMS chart). For each patient, we calculated the absolute difference in the missing fraction in each of the following pairs:

1. CDMS-associated transcript of dictation VS usual care transcript of dictation

2. CDMS chart VS CDMS-associated transcript of dictation

3. CDMS chart VS usual care transcript of dictation

All patients received care from 1 of 3 providers. We used Generalized Estimating Equations (GEE) [26] to perform our comparisons. GEE allowed us to construct multi-level linear models with a structure that takes into account similarity between records created by the same provider. We measured this similarity using the Intraclass Correlation Coefficient (ICC) [26]. The ICC ranges from 0 to 1; values close to 0 mean that there is little relationship between the records of the same provider while values closer to 1 suggest a strong relationship. An ICC of 1 indicates that completeness for a given type of record is constant for records created by the same provider, making the effective sample size of the study 3 instead of 31. We performed all analyses using the STATA Statistical Software package, version 11.2 [27].

## Results

**Figure 5:**
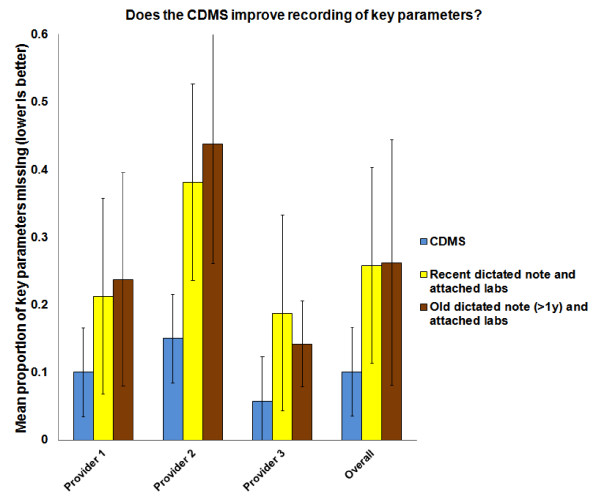
**Does the CDMS improve recording of key parameters?** Key parameters were missing less often from the CDMS chart than from dictated notes. Error bars indicate 95% confidence intervals

Table 1 describes the data; Table 2 presents results of the statistical comparisons and, along with Figure 5, summarizes missing fractions corresponding to each record type overall and by provider.

**Table 1:** Descriptive statistics

Group	N	“Missing” fraction	
		Mean (SEM)%	95% CI
**Usual-care note**	**30**	**26.25 (3.32)**	**19.47, 33.03**
*Provider 1*	10	23.75 (5.00)	12.44, 35.06
*Provider 2*	9	43.75 (5.89)	30.16, 57.34
*Provider 3*	11	14.20 (1.90)	9.97, 18.44
**CDMS-associated note**	**31**	**25.81 (2.60)**	**20.48, 31.12**
*Provider 1*	10	21.25 (2.98)	14.52, 27.98
*Provider 2*	10	38.13 (4.00)	29.07, 47.18
*Provider 3*	11	18.75 (4.04)	9.74, 27.76
**CDMS chart**	**31**	**10.08 (1.18)**	**7.66, 12.50**
*Provider 1*	10	10.00 (1.02)	7.69, 12.31
*Provider 2*	10	15.00 (1.38)	11.87, 18.13
*Provider 3*	11	5.68 (2.30)	0.55, 10.81

CDMS, Chronic disease management system; SEM, Standard error of the mean; CI, Confidence interval.

**Table 2:** Comparison results

Group	N	Absolute difference in“Missing” fraction	
		Mean Δ (SEM*)%	95% CI*
**CDMS-associated note – CDMS chart**	**31**	**15.75 (2.22)**	11.39, 20.11**
*Provider 1*	10	11.25 (3.20)	4.01, 18.49**
*Provider 2*	10	23.13 (3.85)	14.42, 31.83**
*Provider 3*	11	13.07 (4.08)	3.97, 22.16**
**Usual-care – CDMS chart**	**30**	**16.47 (3.02)**	10.55, 22.41**
*Provider 1*	10	13.75 (5.65)	0.96, 26.54**
*Provider 2*	9	29.17 (6.25)	14.75, 43.58**
*Provider 3*	11	8.52 (3.29)	1.18, 15.86**
**Usual-care note – CDMS-associated**	**30**	**0.82% (3.91)**	**−8.48, 6.84**
*Provider 1*	10	2.50 (4.95)	−8.69, 13.69
*Provider 2*	9	5.56 (6.46)	−9.34, 20.45
*Provider 3*	11	−4.55 (4.62)	−14.85, 5.76

*Estimated assuming an exchangeable covariance structure [26]; **p ≤ 0.05; CDMS, Chronic disease management system; SEM, Standard error of the mean; CI, Confidence interval.

The CDMS chart outperformed the CDMS-associated dictated note by a substantial margin (mean missing fraction, 10.1%, 95% CI [7.7% to 12.3%] vs. 25.8%, 95% CI [20.5% to 31.1%], respectively; absolute difference, 15.8%, 95% CI [11.4% to 20.1%]). It similarly outperformed the usual-care dictated note (mean missing fraction, 26.3%, 95% CI [19.5% to 33.0%]; absolute mean difference, 16.5%, 95% CI [10.6% to 22.4%]). However, there was no difference between the usual-care dictated note and the CDMS-associated dictated note (absolute mean difference, 0.8%; 95% CI, −8.5% to 6.8%).

**Figure 6:**
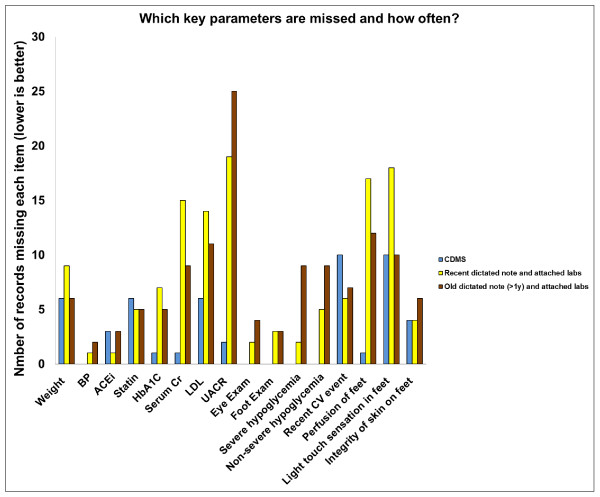
**Which key parameters are missed and how often?** CDMS, Chronic disease management system; BP, Blood pressure; ACEi, Angiotensin Converting Enzyme inhibitor; ARB, Angiotensin II Receptor Blocker; HbA1C, Glycosylated haemoglobin; Cr, Creatinine; LDL, Low density lipoprotein cholesterol; UACR, Urinary albumin to creatinine ratio; CV, Cardiovascular

Figure 6 presents the number of times each of the 16 items was considered missing across the 3 record types. We did not conduct statistical tests to determine the greatest contributors to the missing fraction, but serum creatinine, UACR, hypoglycemia, and perfusion of feet appear to contribute most often.

The ICC for the difference in missing fraction between CDMS charts and CDMS-associated dictation transcripts created by the same practitioner was 0.15 (95 CI, 0.00 to 0.55), 0.25 (0.00 to 0.75) for the difference in missing fraction between CDMS charts and usual care transcripts, and 0 (0.00 to 0.20) for the difference in missing fraction between CDMS-associated transcript and usual care transcript. These estimates are very imprecise because only three providers were included in the study.

## Discussion

We discovered that CDMS charts capture information important for the management of diabetes more often than dictated notes created with or without its use. There was no difference between the two types of dictated notes, suggesting that use of the CDMS neither improved nor hindered the quality of the dictated note among diabetes specialists. We chose the target sample size of 30 patients to help us calculate sample size for future studies of this system, and did not explicitly power the study to detect differences in completeness. With so few patients, we cannot dismiss the possibility of positive or negative impact. Indeed, confidence intervals ranged from substantial improvement to substantial decline in completeness of dictated notes with use of the CDMS.

Completeness of records is only a distant surrogate marker of clinical care quality. It is possible that the items in our assessment form were biased toward the CDMS chart. The CDMS may elicit responses to items that the practitioner deems irrelevant for the dictated summary of a particular clinical encounter. For example, an unremarkable foot exam may not be mentioned in the dictation but the practitioner may still indicate in the CDMS chart (which provides a relevant drop-down menu) that it was performed. In this situation, the patient does not gain additional benefit despite having a more complete CDMS chart.

While the dictated notes did not change with CDMS use, storing more complete records in a structured electronic format allows for other quality improvement interventions. For example, the CDMS includes point-of-care, guideline-based, patient-specific decision support for practitioners and tailored self-management support messages intended for patients. A number of systems that engage diabetic patients and their practitioners have demonstrated benefit in randomized trials [8-10,12]. The CDMS is also being enhanced to provide periodic clinician-level performance feedback, which was previously found to improve the quality of care when combined with patient-specific decision support [28].

Providers were happy with the ability to track patients’ progress longitudinally, and with access to past test results and medication profiles. We can expect some benefit to patient health if particular management decisions require that the practitioner refer to a detailed historical care record. Studies much larger and lengthier than are typically feasible with local computerized interventions would be necessary to detect these benefits, which may be rare but still meaningful given the high prevalence of diabetes.

A specialized diabetes clinic appears to lend itself well to the use of an electronic record and disease management system tailored to the needs of diabetes specialists and their patients. Our findings, however, may not be generalizable to primary care, where most diabetic patients are managed. Primary care physicians address a wide range of ailments within a single patient due to multimorbidity [29] and across patients owing to a broad scope of practice. It may, therefore, be difficult for general practitioners to adopt a detailed system for the management of just one disease.

## Conclusions

In this small, retrospective, before–after pilot study, the CDMS chart captured information important for the management of diabetes more often than dictated notes created with or without its use, but we were unable to detect a difference in completeness between notes dictated in CDMS-associated and usual-care encounters. Larger studies will assess the impact of this system on completeness of records and other, better indexes of care quality.

## Endnote

^a^ One usual care dictated note was not available.

## Competing interests

One usual care dictated note was not available.RJS is the owner of Fig.P Software Incorporated, which develops the chronic disease management system discussed in this study. Other authors declare no competing interests.

## Authors’ contributions

PSR designed the study, acquired, analyzed, and interpreted data; drafted and critically revised the manuscript. HCG conceived the study, contributed to the design of the diabetes-specific CDMS and to the design of the study, acquired and interpreted data and critically revised the manuscript. RJS created the diabetes-specific CDMS and critically revised the manuscript. DLH contributed to the design of the diabetes-specific CDMS and this study; acquired data, and critically revised the manuscript. RBH contributed to the design of the diabetes-specific CDMS and this study, provided study supervision, acquired data and critically revised the manuscript. All authors read and approved the final manuscript.

## Pre-publication history

The pre-publication history for this paper can be accessed here:

http://www.biomedcentral.com/1472-6947/12/63/prepub

## References

[B1] Souza NM Sebaldt RJ Mackay JA Prorok J Weise-Kelly L Navarro T Wilczynski N Haynes RB the CCDSS Systematic Review Team Computerized clinical decision support systems for primary preventive care: a decision-maker–researcher partnership systematic review of effects on process of care and patient outcomes Implement Sci 2011 6 87 10.1186/1748-5908-6-87 21824381 PMC3173370

[B2] Roshanov PS You JJ Dhaliwal J Koff D Mackay JA Weise-Kelly L Navarro T Wilczynski NL Haynes RB the CCDSS Systematic Review Team Can computerized clinical decision support systems improve practitioners’ diagnostic test ordering behavior? A decision-maker–researcher partnership systematic review Implement Sci 2011 6 88 10.1186/1748-5908-6-88 21824382 PMC3174115

[B3] Sahota N Lloyd R Ramakrishna A Mackay J Prorok J Weise-Kelly L Navarro T Wilczynski N Haynes RB the CCDSS Systematic Review Team Computerized clinical decision support systems for acute care management: a decision-maker–researcher partnership systematic review of effects on process of care and patient outcomes Implement Sci 2011 6 91 10.1186/1748-5908-6-91 21824385 PMC3169487

[B4] Hemens BJ Holbrook AM Tonkin M Mackay JA Weise-Kelly L Navarro T Wilczynski N Haynes RB the CCDSS Systematic Review Team Computerized clinical decision support systems for drug prescribing and management: a decision-maker–researcher partnership systematic review Implement Sci 2011 6 89 10.1186/1748-5908-6-89 21824383 PMC3179735

[B5] Nieuwlaat R Connolly S Mackay JA Weise-Kelly L Navarro T Wilczynski NL Haynes RB the CCDSS Systematic Review Team Computerized clinical decision support systems for therapeutic drug monitoring and dosing: a decision-maker–researcher partnership systematic review Implement Sci 2011 6 90 10.1186/1748-5908-6-90 21824384 PMC3170236

[B6] Roshanov PS Misra S Gerstein HC Garg AX Sebaldt RJ Mackay JA Weise-Kelly L Navarro T Wilczynski NL Haynes RB the CCDSS Systematic Review Team Computerized clinical decision support systems for chronic disease management: a decision-maker–researcher partnership systematic review Implement Sci 2011 6 92 10.1186/1748-5908-6-92 21824386 PMC3170626

[B7] Holbrook A Pullenayegum E Thabane L Troyan S Foster G Keshavjee K Chan D Dolovich L Gerstein H Demers C Curnew G Shared electronic vascular risk decision support in primary care: Computerization of Medical Practices for the Enhancement of Therapeutic Effectiveness (COMPETE III) randomized trial Arch Intern Med 2011 171 1736 1744 10.1001/archinternmed.2011.471 22025430

[B8] Holbrook A Thabane L Keshavjee K Dolovich L Bernstein B Chan D Troyan S Foster G Gerstein H Individualized electronic decision support and reminders to improve diabetes care in the community: COMPETE II randomized trial Can Med Assoc J 2009 181 37 44 10.1503/cmaj.081272 19581618 PMC2704409

[B9] MacLean CD Gagnon M Callas P The Vermont Diabetes Information System: a cluster randomized trial of a population based decision support system J Gen Intern Med 2009 24 1303 1310 10.1007/s11606-009-1147-x 19862578 PMC2787948

[B10] Christian JG Bessesen DH Byers TE Christian KK Goldstein MG Bock BC Clinic-based support to help overweight patients with type 2 diabetes increase physical activity and lose weight Arch Intern Med 2008 168 141 146 10.1001/archinternmed.2007.13 18227359

[B11] Cleveringa FG Gorter KJ van den Donk M Rutten GE Combined task delegation, computerized decision support, and feedback improve cardiovascular risk for type 2 diabetic patients: a cluster randomized trial in primary care Diabetes Care 2008 31 2273 2275 10.2337/dc08-0312 18796619 PMC2584178

[B12] Peterson KA Radosevich DM O’Connor PJ Nyman JA Prineas RJ Smith SA Arneson TJ Corbett VA Weinhandl JC Lange CJ Hannan PJ Improving diabetes care in practice: findings from the TRANSLATE trial Diabetes Care 2008 31 2238 2243 10.2337/dc08-2034 18809622 PMC2584171

[B13] Augstein P Vogt L Kohnert KD Freyse EJ Heinke P Salzsieder E Outpatient assessment of Karlsburg Diabetes Management System-based decision support Diabetes Care 2007 30 1704 1708 10.2337/dc06-2167 17468357

[B14] Filippi A Sabatini A Badioli L Samani F Mazzaglia G Catapano A Cricelli C Effects of an automated electronic reminder in changing the antiplatelet drug-prescribing behavior among Italian general practitioners in diabetic patients: an intervention trial Diabetes Care 2003 26 1497 1500 10.2337/diacare.26.5.1497 12716811

[B15] Meigs JB Cagliero E Dubey A Murphy-Sheehy P Gildesgame C Chueh H Barry MJ Singer DE Nathan DM A controlled trial of web-based diabetes disease management: the MGH diabetes primary care improvement project Diabetes Care 2003 26 750 757 10.2337/diacare.26.3.750 12610033

[B16] Lobach DF Hammond W Computerized decision support based on a clinical practice guideline improves compliance with care standards Am J Med 1997 102 89 98 10.1016/S0002-9343(96)00382-8 9209205

[B17] Nilasena DS Lincoln MJ A computer-generated reminder system improves physician compliance with diabetes preventive care guidelines Proc Annu Symp Comput Appl Med Care 1995 640 645 8563365 PMC2579172

[B18] Mazzuca SA Vinicor F Einterz RM Tierney WM Norton JA Kalasinski LA Effects of the clinical environment on physicians’ response to postgraduate medical education Am Educ Res J 1990 27 473 488

[B19] Thomas RE Croal BL Ramsay C Eccles M Grimshaw J Effect of enhanced feedback and brief educational reminder messages on laboratory test requesting in primary care: a cluster randomised trial Lancet 2006 367 1990 1996 10.1016/S0140-6736(06)68888-0 16782489

[B20] Derose SF Dudl JR Benson VM Contreras R Nakahiro RK Ziel FH Point of service reminders for prescribing cardiovascular medications Am J Manag Care 2005 11 298 304 15898218

[B21] Sequist TD Gandhi TK Karson AS Fiskio JM Bugbee D Sperling M Cook EF Orav EJ Fairchild DG Bates DW A randomized trial of electronic clinical reminders to improve quality of care for diabetes and coronary artery disease J Am Med Inform Assoc 2005 12 431 437 10.1197/jamia.M1788 15802479 PMC1174888

[B22] Thomas JC Moore A Qualls PE The effect on cost of medical care for patients treated with an automated clinical audit system J Med Syst 1983 7 307 313 10.1007/BF00993294 6619687

[B23] Martin DC Berger ML Anstatt DT Wofford J Warfel D Turpin RS Cannuscio CC Teutsch SM Mansheim BJ Turpin RS A randomized controlled open trial of population-based disease and case management in a Medicare Plus Choice health maintenance organization Prev Chronic Dis 2004 1 A05 15670436 PMC1277945

[B24] Demakis JG Beauchamp C Cull WL Denwood R Eisen SA Lofgren R Nichol K Woolliscroft J Henderson WG Improving residents’ compliance with standards of ambulatory care: results from the VA Cooperative Study on Computerized Reminders JAMA 2000 284 1411 1416 10.1001/jama.284.11.1411 10989404

[B25] Hetlevik I Holmen J Kruger O Implementing clinical guidelines in the treatment of hypertension in general practice. Evaluation of patient outcome related to implementation of a computer-based clinical decision support system Scand J Primary Health Care 1999 17 35 40 10.1080/028134399750002872 10229991

[B26] Zeger SL Liang K-Y Albert PS Models for longitudinal data: a generalized estimating equation approach Biometrics 1988 44 1049 1060 10.2307/2531734 3233245

[B27] StataCorp Stata Statistical Software: Release 11 2009 StataCorp LP, College Station, TX

[B28] Lobach DF Electronically distributed, computer-generated, individualized feedback enhances the use of a computerized practice guideline Proc AMIA Annu Fall Symp 1996 493 497 8947715 PMC2233009

[B29] Fortin M Bravo G Hudon C Prevalence of multimorbidity among adults seen in family practice Ann Family Med 2005 3 223 228 10.1370/afm.272 PMC1466875 15928225

